# A Practice of BLE RSSI Measurement for Indoor Positioning

**DOI:** 10.3390/s21155181

**Published:** 2021-07-30

**Authors:** Ramiro Ramirez, Chien-Yi Huang, Che-An Liao, Po-Ting Lin, Hsin-Wei Lin, Shu-Hao Liang

**Affiliations:** 1 Department of Industrial Engineering and Management, National Taipei University of Technology, Taipei 106344, Taiwan; t109379402@ntut.edu.tw (R.R.); jayhuang@mail.ntut.edu.tw (C.-Y.H.); 2 Department of Mechanical Engineering, National Taiwan University of Science and Technology, Taipei 106335, Taiwan; m10803146@mail.ntust.edu.tw (C.-A.L.); potinglin@mail.ntust.edu.tw (P.-T.L.); m10903k01@mail.ntust.edu.tw (H.-W.L.); 3 Industry 4.0 Implementation Center, National Taiwan University of Science and Technology, Taipei 106335, Taiwan

**Keywords:** BLE, RSSI, IPS, trilateration, modification coefficient, Kalman filter

## Abstract

Bluetooth Low Energy (BLE) is one of the RF-based technologies that has been utilizing Received Signal Strength Indicators (RSSI) in indoor position location systems (IPS) for decades. Its recent signal stability and propagation distance improvement inspired us to conduct this project. Beacons and scanners used two Bluetooth specifications, BLE 5.0 and 4.2, for experimentations. The measurement paradigm consisted of three segments, RSSI–distance conversion, multi-beacon in-plane, and diverse directional measurement. The analysis methods applied to process the data for precise positioning included the Signal propagation model, Trilateration, Modification coefficient, and Kalman filter. As the experiment results showed, the positioning accuracy could reach 10 cm when the beacons and scanners were at the same horizontal plane in a less-noisy environment. Nevertheless, the positioning accuracy dropped to a meter-scale accuracy when the measurements were executed in a three-dimensional configuration and complex environment. According to the analysis results, the BLE wireless signal strength is susceptible to interference in the manufacturing environment but still workable on certain occasions. In addition, the Bluetooth 5.0 specifications seem more promising in bringing brightness to RTLS applications in the future, due to its higher signal stability and better performance in lower interference environments.

## 1. Introduction

The adoption of an indoor positioning system (IPS) represents an important technological upgrade for different institutions, such as exhibition centers, museums, retail stores, underground transportation facilities, and healthcare centers, among others. The implementation of IPS solutions can provide location data in closed environments, which can be used for indoor navigation, a foot traffic analysis, or enhanced retail experiences. In addition, integration with autonomous vehicles in factories and production lines for the industry sector is currently under development, due to its higher system requirements [1]. The RF-based IPS working with the Received Signal Strength Indicator (RSSI) has been one of the most popular technologies for decades [2]. In practice, there are many types of RF-based technologies that can apply to IPS development with RSSI, including Bluetooth Low Energy (BLE), WIFI [3], ultra-wideband (UWB) [4], the 5G cellular network [5], and many others.

Furthermore, most smartphone-embedded BLE antennas today make the BLE technology take advantage of other RF-based technologies. It benefits from the development of applications related to interacting with humans. Additionally, the entire mature Bluetooth technology ecosystem contributes to the deployment of large-scale and various applications. Meanwhile, BLE technology has continued improving its signal stability and propagation distances, as BLE 5.0 demonstrated. On the other hand, the academia and industry have completed many significant studies relevant to indoor positioning systems (IPS) and real-time location systems (RTLS) in the past, mostly focusing on the mass market of pedestrian applications. However, special attention should be given to IPS solutions suitable for working environment conditions, such as factories [6].

Regarding the importance and volume of IPS applications in the market, the indoor positioning system market size could reach USD 17.0 billion by 2025, according to the market report of Markets&Markets [7]. Moreover, the outdoor locating service with GPS has increased in our daily life, with tools such as Google maps, taxi fleet management, 911 emergency application [8], and many applications relevant to location-based services (LBSs) [9]. Therefore, the increasing use of location services, the improvement and evolution of Bluetooth technologies, and the market predictions performed by specialists make us believe the demands for location positioning will continue to grow, shifting from outdoor to indoor applications in the near future.

IPS RF-based applications are mainly classified into three categories: (1) Range-based positioning, (2) Fingerprinting, and (3) Proximity-based positioning [10]. Currently, IPS applications are increasing their presence on the market due to two main factors, the improvement of the electronic circuits and a significant price reduction, both enabled by the increasing demand of Bluetooth solutions [11]. In addition, innovative methods in direction-finding applications such as AoA (angle of arrival) and AoD (angle of departure) over RSS measurements can achieve higher location accuracy [12,13,14]. Unfortunately, there is not a standardized indoor positioning system solution. This issue is a major concern for enterprises, government agents, medical institutes, or other organizations willing to introduce IPS solutions. Under this situation, reviewing and understand the positioning technologies using RSSI can be essential for all relative technology developments.

It is important to understand that RF-based indoor positioning systems are especially susceptible to interference from the peripheral environment. Special attention should be given to the influence of metal objects, solid barriers (such as walls or furniture), and signal interference from Wi-Fi radio signals [15]. Due to this level of complexity, multiple related studies are carried out every year by both academic and industrial researchers in order to explore, improve, and suggest different IPS solutions. Currently, RSSI measurements cannot achieve positioning accuracy within a meter-level without the corresponding algorithm [16].

In order to understand the whole IPS paradigm, this work will be mainly focused on Range-based positioning applications, which are specially recommended for short-range environments. The research will be divided into three main points: signal measurements, analysis methods, and results. The purpose is to explore the factors in IPS development, such as antenna relative orientation, steady-signal acquisition, spatial geometric issues, environmental influences (obstacles), and appropriate algorithms. Two types of BLE microcontrollers were selected for this study, proposing an interconnection architecture to prevent data congestion in signal collection.

In practice, we adapted an active positioning model where the mobile device (scanner) can change its position, collecting multiple RSSI values from the detected BLE beacons that surround the experimental areas. After detection, the mobile device (scanner) packs, transports, and delivers the data to a remote server, where the measured values will be stored. In contrast, each beacon will be fixed in a well-known point in space (X-Y coordinates). This setup refers to the transmitter-based arrangement [17]. The RSSI data of the beacon is discrete in the scale of distance and needs to exploit the Trilateration and Kalman filtering to find the precise positioning [18].

In the end, centimeter-level positioning accuracy of approximately 8–10 cm was achieved in a relatively low interference environment. A proposed interconnection architecture provided data transmission among beacons, scanners, and databases with less latency concern. In addition, it is essential to highlight that the Bluetooth 5.0 component presented a better performance than the previous version in this experiment. Applying the IPS system in the factory environment requires continuous effort to study component settings and algorithms.

## 2. Materials and Methods

This section describes the materials and methods used in this study, including components, measurement, and analysis methods. The components portion explains the specifications and characteristics of the components used in experiments and, also, the data transmission path, protocols, and hardware architectures. The measurement portion contains three measurement plans: RSSI–distance conversion, Multi-beacon in-plane, and diverse directional in geometry measurements. Finally, the analysis method portion depicts the data process in the calculations, supported by the Signal propagation models, Trilateration, Modification coefficients, and Kalman filter equations. The main subjects conducted in this study are listed in Table 1.

**Figure 1:**
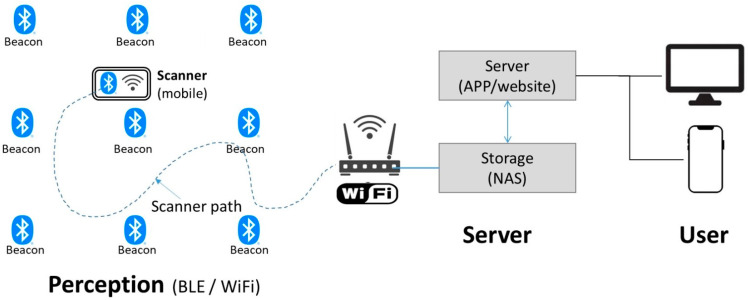
System schematic.

The IPS system consists of perception, server, and user interface segments, as shown in Figure 1. Beacons transmit RF signals to the scanner (receiver) via Bluetooth in the perception segment. Simultaneously, the scanner passes the RSSI of the received signals to the server segment. The server segment contains the storage (NAS, network-attached storage) and application server (application and website). The application program first reads the RSSI data from the database and, after performing its calculation, stores the positioning coordinates of the calculation result back to the database.

### 2.1. Components

Two specifications of Bluetooth components, including BLE 4.2 and BLE 5.0, are used as beacons and scanners to evaluate the signal stability and effectivity in experiments. As the Bluetooth Special Interest Group (SIG) announced, BLE 5 significantly increased the range, speed, and broadcast messaging capacity compared to the previous BLE 4 [19]. It is still necessary to verify the signal attribute difference in the field between them. Furthermore, since BLE 4 has been developed and used for an extended period, there are many application deployments. Therefore, these two, new and old, have incredible opportunities to work in pairs in the application field.

The RSSI-based indoor positioning system should contain transmitters and receivers, known as a beacon and scanner, respectively. There are two types of configurations for the deploy beacon and receiver: tag-based [20] and beacon-based [21]. This study used the beacon-based configuration, with the beacons placed in a fixed position, and the scanner acted as a moving element.

#### 2.1.1. Components Specification

The selected components for the experiment are both microcontroller units (MCU): Arduino Nano 33 and Linkit 7697. Their main features are shown in Table 2. The selection of MCUs relies on three main factors: standardization, customization, and flexibility.

Firstly, the multiple offer of Beacons in the market is enormous, from companies such as BlueCats [22], Estimote [23], or Kontak.io [24] providing commercial versions of beacons. To simplify this issue, our approach focused on the use of similar hardware for transmission and reception in order to guarantee a more standard procedure. Secondly, the customization of critical parameters (advertising the interval and scanning times) from emitters and receivers is crucial to generate a significant amount of data, guarantee an appropriate sampling rate, and control the overall system latency. Finally, the selected hardware (Linkit 7697) offers both Wi-Fi and Bluetooth connectivity, and it is able to support the MQTT and CoAP protocols, facilitating the interconnectivity and providing flexibility on the way to achieve this interconnection between the receiver (scanner) and the database.

Based on the technical specifications, the transmitting segment in dBm (decibel relative to one milliwatt) for Linkit 7697 is slightly higher than Arduino Nano. On the other hand, BLE 5.0 can perform a little better sensitivity in the receiving segment: −103 dBm at the long-range mode and −95 dBm at the low-energy mode. To make the experimental data clearer and readable, Linkit 7697 will work as the scanner. The scanners were used to collect the transmitting signal of beacons Arduino nano 33 and Linkit 7697, respectively.

**Figure 2:**
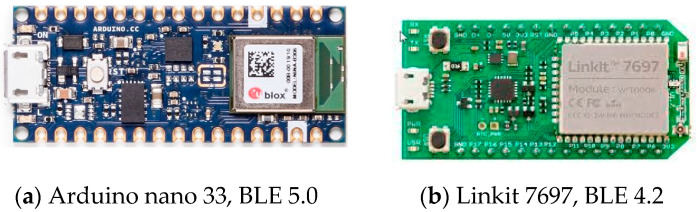
The appearance of Arduino nano 33 and Linkit 7697.

The appearances of those two components are shown in Figure 2a,b: Arduino nano 33, BLE 5.0 and Linkit 7697, BLE 4.2. Both use a ceramic antenna design, so there is no antenna rod in appearance. Generally, the flat antenna makes it more convenient for conducting a test with less consideration toward directional issues. However, in practice, the antenna orientation still causes a deviation of the signals among the devices.

#### 2.1.2. Antenna Orientation Issues (Distance Limitation)

As mentioned previously, both devices use a ceramic antenna for emitting and receiving signals. The ceramic antenna offers several advantages, such as multiple configurations, a small size, less sensitivity to components, and less environmental noise. However, its main shortcoming is the slight lack of performance compared to the PCB trace antenna [25].

**Figure 3:**
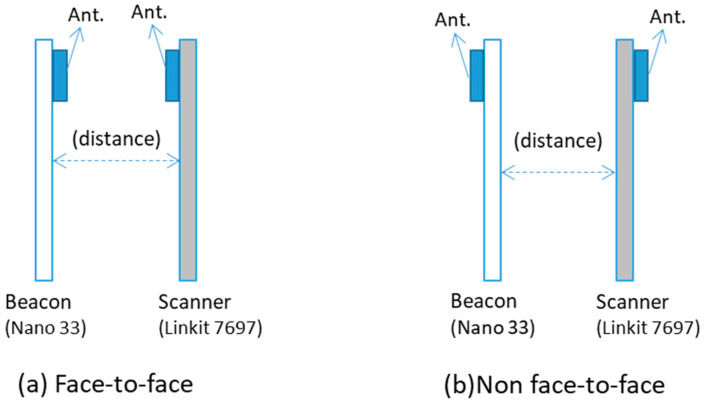
Antenna orientation: (**a**) face-to-face and (**b**) not face-to-face.

Additionally, the antenna can only be on one side of the circuit board. Thus, to understand the influence of antenna orientation on signal reception, two positions were selected for testing, “face-to-face” and “back-to-back”, as shown in Figure 3a,b.

The orientation influences the RSSI between the beacon and scanner, regardless of their model. We conducted measurements with Arduino nano 33 as a beacon and Linkit 7697 as a scanner to prove this effect. The results are shown in Table 3.

According to the sensitivity of the scanner, as shown in Table 2, signals under −94 dBm cannot be considered as a correct lecture. It is possible to observe that the back-to-back antenna configuration caused a significant impact on the strength of the reception or RSSI even at a short distance, as shown in Table 3. On average, the back-to-back orientation decreases 19 dBm in comparison to the face-to-face antenna orientation. This difference can generate significant location positioning errors. For this reason, all measurements conducted in this study remained in a particular orientation, face-to-face, as mentioned in Figure 3a.

#### 2.1.3. Floor Plan (Experimental Site)

The experiments of this work were performed at the Industry 4.0 Implementation Center from the National Taiwan University of Science and Technology (NTUST). The center is an educational shared demo factory that contains multiple spaces, such as a workshop, tool room, laboratory, meeting room, and classroom.

**Figure 4:**
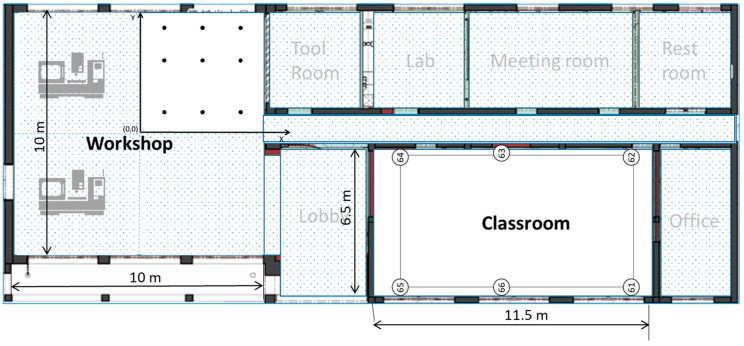
Experiment field layout (workshop and classroom).

The main experiment fields include two different environments: a classroom and workshop, as shown in Figure 4. The classroom space is regarded as a relatively low-noise environment, with dimensions of 11.5 m by 6.5 m. On the other hand, the workshop is regarded as a relatively noisy environment, with dimensions of 5 m by 5 m (one-quarter of the space). Besides the size limitation, the workshop space contains heavy machinery around its surroundings, providing a closer approach to real-life factory environment conditions.

An internal numerical system was imposed to differentiate the hardware components in both environments. Thus, for the classroom, Arduino nano 33 has numbers #51–56 (6 pieces) and Linkit 7697 has numbers #61–66, respectively. Similarly, Arduino Nano33 has been assigned the numbers #71–79 (9 pieces) and Linkit 7697 #81–89 (9 pieces) at the workshop, as shown in Table 4.

#### 2.1.4. Interconnection

Positioning systems such as real-time locating systems (RTLS) are time-sensitive and require a stable positioning update interval. Therefore, choosing an appropriate and effective communication architecture is very important to reduce data lagging or call latency.

Following the TCP/IP architecture, it is possible to decompose the whole IPS paradigm into four main layers: link, internet, transport, and application [26]. First, the link layer defines both the physical and data link protocols, such as Bluetooth, 802.11 b/g/n, and MAC. Microcontrollers (MCU) and General Purpose Units (GPU) are in charge of this layer. Second, the internet layer relies on the Internet Protocol (IP) to pack and deliver higher layers. For example, access point (AP) devices support IPs on both versions (IPv4 and IPv6), using ports 1027 and 3784, respectively. Third, the transport layer relies on the Transport Control Protocol (TCP), and routers and firewalls handle TCP packages. Finally, the application layer hosts Message Queuing Telemetry Transport (MQTT), working primarily on TCP/IP, using ports 1883 and 21883, respectively. The Table 5 interconnection model and protocols are conducted in the following experiments.

Servers and storage components rely on the use of the MQTT protocol for interconnections. The package structure follows a JavaScript Object Notation (JSON) stored in MongoDB, a NoSQL database. Implementation of the Bluetooth stack can vary according to the type of hardware. Therefore, it is possible to have a General Purpose Bluetooth Stack (BlueZ) [27] or Embedded system implementations (BlueMagic) [28]. The selected device used for research on this topic, MT7697, works with a Qualcomm version of BlueMagic (MediaTek) [29].

General-purpose data collection systems monitor the value of the signal and export single values in comma-separated values (CSV) stored locally every 500 ms [30]. Using embedded systems to harvest data with the proposed protocol architecture allows generating over 200,000 samples for two hours or an average of 32 ms per sample. Thus, the suggested framework to collect, pack, transport, and deliver data to the database provides a solid base to develop IPS solutions to generate a significant amount of data in a shorter period, improving the Overall Operation Effectiveness (OOE).

### 2.2. Measurement

The measurements consist of three stages: RSS-distance conversion (one-dimension scale), multi-beacon in-plane (two-dimension scale), and diverse directions in a geometric spatial scale (three-dimension scale). These three stages of measurements are related to each other; the initial measurement result affects the subsequent measurements and calculations.

**Figure 5:**
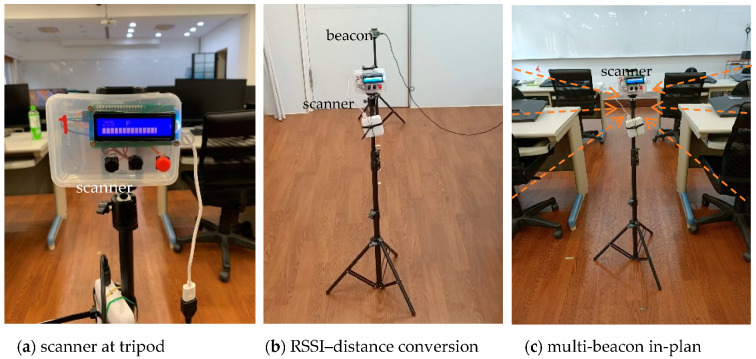
Measurement configurations in the field.

Figure 5 depicts the measurement configuration in the field for RSSI–distance conversion multi-beacon in-plan measurements. Figure 5a shows the scanner mounted on the tripod. The scanner orientation remained when performing measurement tasks for the RSSI–distance conversion and multi-beacon in-plan (at classroom), as shown in Figure 5b,c.

**Figure 6:**
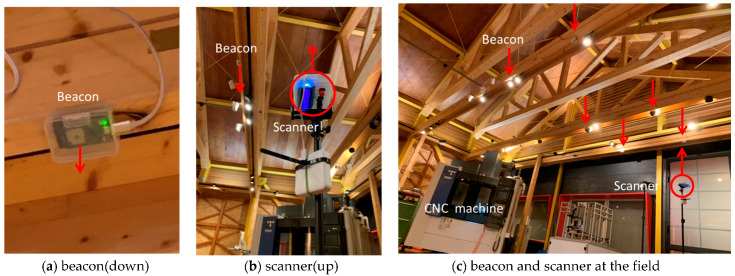
Measurements scene at the workshop.

Figure 6 denotes the measurement scene at the workshop. The antenna orientation of the beacons faces the ground, as shown in Figure 6a; on the contrary, the antenna on the scanner is facing up, pointing to the beacons, as shown in Figure 6b. The beacons are fixed on the beam of the structure, and the mobile scanner mounted on a tripod is made to move around the different measuring points, as shown in Figure 6c.

#### 2.2.1. RSSI–distance Conversion

**Figure 7:**
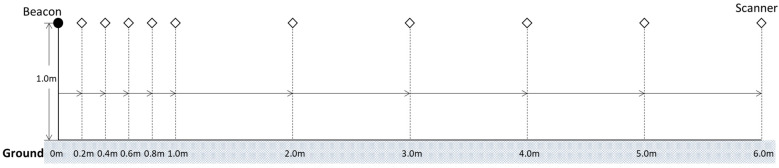
Distance between the beacon and scanner in the measurements.

RSSI–distance conversion (one-dimensional scale) measurements establish the scale between RSSI and distance, which becomes the benchmarks for the data collection and following calculations. The measurement and calculation steps refer to the Google Android Library guidelines to conduct the regression and prediction [31]. Based on this reference, several measurement points for the RSSI–distance conversion measurements are fixed, as shown in Figure 7.

When performing the measurements, the beacon stays steady at the original, and the scanner moves gradually by 0.2 m until it reaches 6 m, scanning 1000 samples at each point. The scene refers to Figure 5b. When the measurements extend up to 12 m, some of the measured signal strength is already weaker than the specifications of the component sensitivity; therefore, it is not indicated in the figure.

#### 2.2.2. Multi-Beacon in-Plane

The purpose of multi-beacon measurements is to explore the difference of RSSI when the scanner is dealing with multiple beacons, so-called Multi-beacon in-plane measurements. Compared with the previous measurements (RSSI–distance conversion), the scanner must simultaneously scan the signals emitted by multiple beacons. Beacon wireless signal interference and radio reflection from the wall are two main problems to consider in this situation. However, radio wave interference-related issues will not be discussed in this section. Instead, the focus will be given to data collection and data optimization.

**Figure 8:**
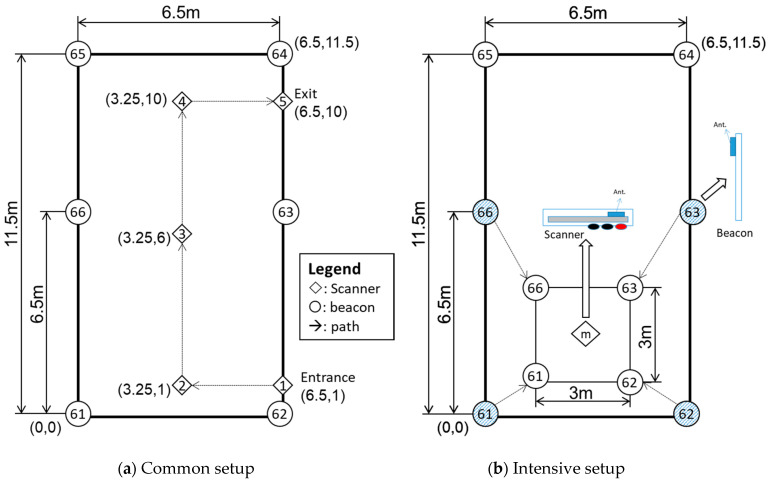
Dimensions of the layout in the multi-beacon scenario.

First, all the beacons and scanners remained one-meter height from the ground. Multi-beacon measurements were performed in a relatively less noisy environment, the classroom, in an 11.5 m by 6.5 m space, as shown in Figure 8. There were two different configurations of the beacon layout: Common setup and Intensive layout, as Figure 8a,b shows. The Intensive layout mainly considers the limitations of the performance of the components; the maximum sensitivity of −94 dBm refers to Table 2.

During the measuring procedure, the scanner starts from the first point (P1) (diamond symbol), located at the lower-right corner of Figure 7. Its coordinates (6.5, 1) are relative to the original coordinates (0,0), given in meters. The scanner moves from P1 towards P5 to complete the measurements, as shown in Figure 8a. Since the scanner has to scan six beacons simultaneously, the distance to each beacon in the field is different. Additionally, the timestamp of each scan will not be the same; in conclusion, the RSSI data quantity collected from each beacon is not even. Roughly, the scanner takes about 32 ms to complete a piece of RSSI data from beacon to scanner and save it to the database. The scanner maintains the same direction when it moves in the path.

During the measurements, when the distance between beacon and scanner increased, the signal strength became weaker. In the Common setup, the distances of multiple points are outside of the limit of sensitivity. For example, beacons #64 and #65 might not transmit sufficient RSSI to point 2, because the distance is over 10 m. Most indoor positioning systems usually recommend to place the beacon on a wall because of the convenience of installation [32]. Nevertheless, the effect of wall reflection on indoor wireless locations based on RSSI has been verified by Fan et al. [33]. Therefore, it is possible to avoid refraction and improve the signal strength when the beacon is separated from the wall.

Combining the two previous arguments, we decided to conduct the Intensive mode. The Intensive mode moved beacons 61, 62, 63, and 66 to a 3 m × 3 m square and placed the scanner at point m (square center), as shown in Figure 8b. The differences between the Common setup and Intensive setup positioning results will be significant.

#### 2.2.3. Diverse Directions in Geometric Spatial

Diverse directions in geometric spatial measurements change the relative orientation of the beacons and scanner antennas into a three-dimension space. As a result, the relative orientation of the beacons and scanners is a bit close to the face-to-face configuration plus various angles, and the measurement scene refers to Figure 6.

- Configuration and path

**Figure 9:**
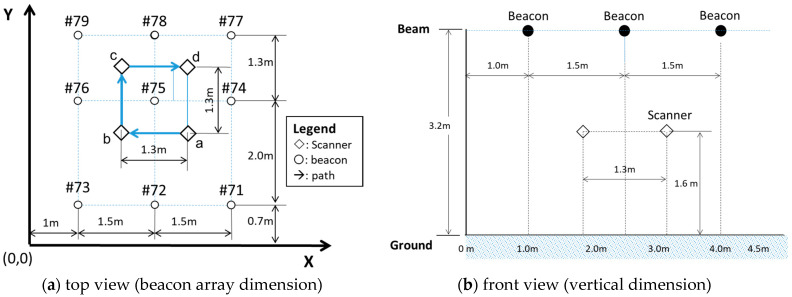
Beacon configuration and scanner path.

This experiment utilized nine beacons to form a beacon array. Figure 9 shows the positions of the beacons and the path of the scanner movement. The distance between the beacons was a 1.5-m pitch in the x-axis and 0.7 m, 2.0 m, and 1.3 m, respectively, in the y-axis direction, as shown in Figure 9a, the top view of the beacon array. The positions of the beacons numbered #71–79 means using Nano 33. Points a, b, c, and d represent the measuring points, in which the scanner follows the arrow path to conduct the measurements, scanning 1000 samples at each point.

Figure 9b shows the configuration of the beacons and scanners in the vertical direction, the beacon is 3.2 m, and the scanner is 1.6 m in height, respectively. The heights of the beacons and scanner may be variable in factories and warehouses. This setting is based on the restrictions of the building itself and the shipping standards of the goods (DHL standard pallet height).

- Three-dimension in space

**Figure 10:**
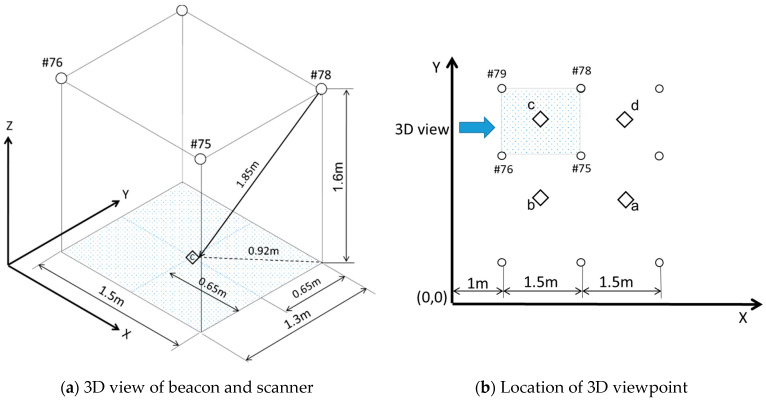
Geometric dimension—3D view.

The distances of the beacons and scanners in the three-dimensional space are different from in the same plane. Figure 10 shows the relative positions between beacons #75, #76, #78, and #79 and the scanner at point c in 3-dimensional coordinates in space. The actual distance between the scanner at point c and beacon #78 by calculations based on geometry is 1.85 cm. In the same manner, geometric calculations derive the distance between the scanner locations a, b, c, and d to the beacons, as shown in Table 6.

The indoor positioning defines the location regarded as the X-Y coordinates of the space. Therefore, the actual distance between the beacon and the scanner denotes geometric dimensions in the 3D view, as shown in Figure 10a,b. Nevertheless, the RSSI, signal strength, is related to the actual distance in 3D space; thus, the distance in the x-y plane must be converted by the Trigonometric function. As a result, the distance of each beacon correlative to the points a, b, c, and d (scanner stay points) was calculated, as shown in Table 6.

### 2.3. Analysis Methods

The analysis methods include the Signal propagation model, Trilateration, Modification coefficients, and Kalman filter algorithm. The following subsections will present the main traits of each method.

#### 2.3.1. Signal Propagation Model

Distance estimation from the RSSI might refer to some models, such as the Log-Distance Path Loss model [34], International Telecommunication Union (ITU) model [35], and the empirical model suggested by Cantón Paterna et al. [18]. The model of RSSI corresponding to the distances based on the signal–distance equation, Formula (1), is expressed as the signal ratio (R/T) between the current RSSI (R) value of the emitter (beacon) against a reference value of an external device at one meter from the receiver (T). The other parameters are the environment constants that can be obtained empirically by measuring the RSSI at multiple distances [36].

(1) $$d=\alpha +\beta \left(\;\frac{R}{T}\;\right)+\gamma ,$$

where *α* and *β* are the regression variables of the power regression between the signal ratio (R/T) and the actual distance; the value of *γ* can be estimated as the difference between the actual distance and the estimated distance (Formula (2)).

(2) $$\gamma =1-\alpha +\beta \left(\;\frac{R}{T}\;\right)$$

In practice, different combinations of transmitter and receivers can affect the results of the RSSI data measurements. Even if the product of the same model is used in the actual positioning calculations, the RSSI value must be measured and verified with different combinations of transmitters and receivers. The receiver’s exclusive distance conversion formula can reduce the subsequent positioning error.

#### 2.3.2. Trilateration

Triangulation and Trilateration are two of the most common indoor positioning methods, using geometric characteristics between devices and positioning points. Triangulation uses geometric characteristics of the triangle formed in a two-dimensional environment. However, in practice, this estimation often generates errors.

In contrast, Trilateration [37] measures the distance between a signal point and the observation point using the Least Square Method (LSM), Time Difference of Arrival (TDOA), and measured signal strength (RSSI). It offers a more accurate position in comparison to the triangulation capabilities. In addition, LSM can minimize the data error in numerical calculations if the system error follows a random normal distribution (RND).

Assuming the position of a point **P** is denoted as P=[X,Y], the *i*th position of the sensor is given as $S_{i}=\left[S_{i,1},S_{i,2}\right]$:

(3) $${\|S}_{i}-P\|=L_{i}$$

or

(4) $$S_{i,1}^{2}-2S_{i,1}X+X^{2}+S_{i,2}^{2}-2S_{i,2}Y+Y^{2}=L_{i}$$

For i=1,…,N, where N is the number of sensors. When N=3, it is a trilateration method; N>3 is called a multilateration method. The quadratic terms of $X^{2}$ and $Y^{2}$ in the above equation could be eliminated by subtracting the *j*th equation from the ith one, yielding:

(5) $$\left[\begin{array}{c} \begin{array}{c} \left(L_{1}-S_{1,1}^{2}-S_{1,2}^{2}\right)-\left(L_{2}-S_{2,1}^{2}-S_{2,2}^{2}\right)\\ \left(L_{1}-S_{1,1}^{2}-S_{1,2}^{2}\right)-\left(L_{3}-S_{3,1}^{2}-S_{3,2}^{2}\right) \end{array}\\ \vdots \\ \left(L_{N-1}-S_{N-1,1}^{2}-S_{N-1,2}^{2}\right)-\left(L_{N}-S_{N,1}^{2}-S_{N,2}^{2}\right) \end{array}\right]=\left[\begin{array}{c} \begin{array}{c} -2S_{1,1}+2S_{2,1}\\ -2S_{1,1}+2S_{3,1}\\ \vdots \end{array}\\ -2S_{N-1,1}+2S_{N,1} \end{array}\begin{array}{c} \;\;\begin{array}{c} \;-2S_{1,2}+2S_{2,2}\\ \;-2S_{1,2}+2S_{3,2}\\ \vdots \end{array}\\ \;\;\;-2S_{N-1,2}+2S_{N,2} \end{array}\right]\cdot \left[\begin{array}{c} X\\ Y \end{array}\right]$$

The above equation can be solved by the Least Squares Approximation (LSA).

In practice, the trilateration method requires three main steps: firstly, establishing a horizontal triangular grid between the measurement points; secondly, calculating the length of each side through the signal collected by the sensor; and finally, calculating the point according to the relationship between the geometric principle of the triangle.

#### 2.3.3. Modification Coefficients

**Figure 11:**
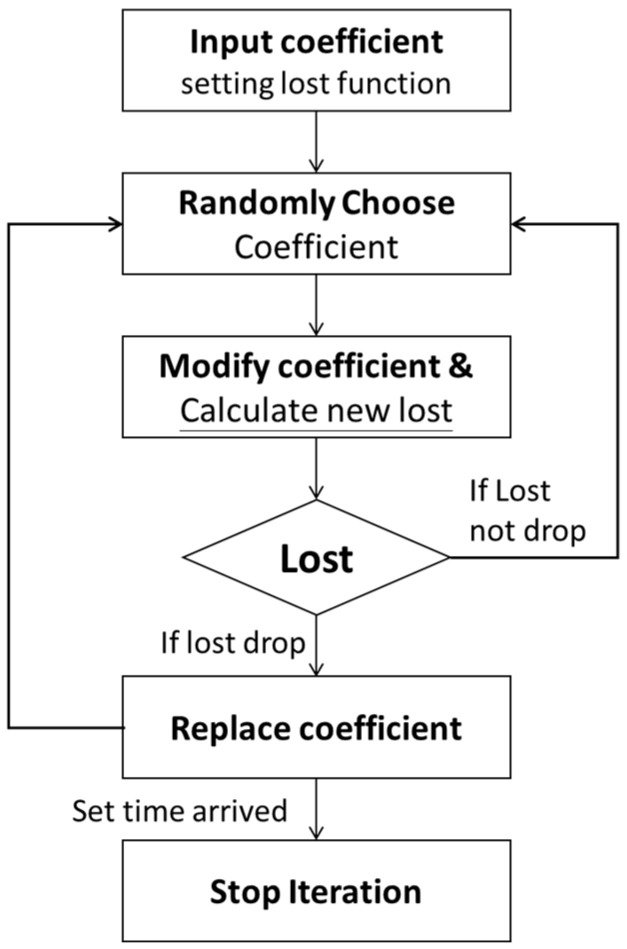
Modification coefficients workflow.

In the positioning calculations, random iteration is used to modify the coefficient. The Random Walk (RW) explains many processes’ observed behaviors and applies to engineering and many scientific fields, including ecology, psychology, and computer science. Thus, it serves as a fundamental model for recorded stochastic activity [38]. The execution process of Modification coefficients is shown in Figure 11.

#### 2.3.4. Kalman Filtering

Kalman filtering is a well-known method to help reduce the impact of wrong measurements on the system, and it can avoid incoherent computation of the location. Mismeasurements of RSSI could lead to significant errors in location positioning, according to the study by Cantón Paterna et al. [18]. A New Kalman filter-based algorithm to improve indoor positioning was proposed by Nabil et al. [39]. Moreover, an extended Kalman filter method was developed to improve indoor positioning accuracy by Lee et al. [40]. Currently, there are multiple Kalman filters used in indoor positioning to enhance location positioning.

This research is based on indoor short-distance positioning, focusing on measuring the signal strength (RSSI), distance estimation, trilateration method (Trilateration), and modification coefficients optimization. We reviewed the comparative methods and developed a new type of Kalman filter algorithm for subsequent experiments. We did not plan apply a Kalman filter on the RSSI dataset, since the current Kalman filter was tested for its effectiveness.

## 3. Results

This section describes the measured data and its results regarding the RSSI-conversion, multi-beacon in-plane, and diverse directions in geometric spatial measurements. The initial measured data must be statistically analyzed before it can be used for calculations with the materials and methods previously mentioned.

### 3.1. RSSI-Conversion

RSSI-conversion is the essential part of all experiments, because subsequent calculations are related to it like a scale. A Bluetooth signal is easily affected by the terrain and obstacles, resulting in multiple problems such as attenuation, interference, and reflection. Location positioning needs a specific value or a mean dBm in RSSI–distance conversion for further calculations, such as the Trilateration or Modification coefficients in this study. Through statistical methods, some discrete data can obtain a more definite value, using the median value of the dataset.

Table 7 presents the signal strength corresponding to the distance, and it is the benchmark to use for positioning calculations. The measurement range is 0.2–10 m, using Arduino Nano 33 and Linkit 7697. The data in Table 7 determined by the regression coefficient and outlier data are explained in the following paragraph.

- Regression coefficient

**Figure 12:**
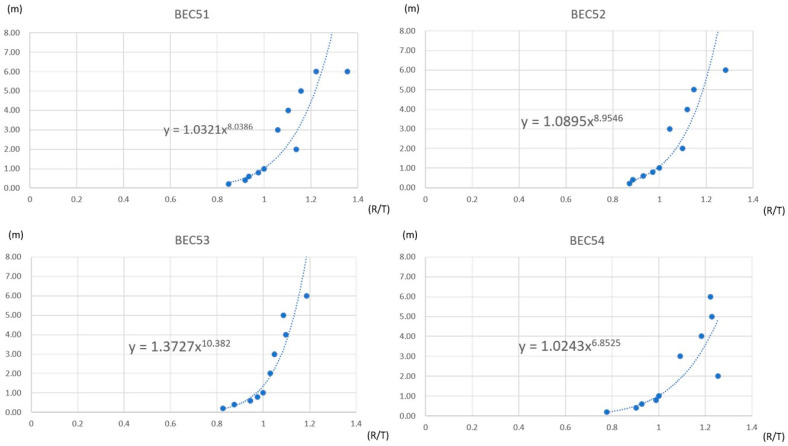
Regression coefficients for different beacons (BEC51–BEC54).

The measured RSSI of each beacon can fluctuate considerably regarding the mass production tolerance, measurement environment, and antenna direction, among other factors. The regression coefficients analyzed individual differences among the beacons according to the signal propagation model, Formula (2) in Section 2.3.1. Here, the formula notation transformed to D = a·(R/T)^b^ + c to fit in the computing format, where D represents the converted distance; R refers to the RSSI strength; T is the RSSI at a 1-m distance; and a, b, and c are the coefficients. Figure 12 shows those regression coefficients for different beacons (BEC51–BEC54) in the different distances, 0–8 m.

- Box plot and outlier data

The outlier signal values can lead to positioning errors so that we must define a threshold to filter out the signal with a significant deviation. RSSI −94 dBm can be an appropriate threshold regarding the sensitivity of the components (Nano 33, −95 dBm; Linkit 7697, −94 dBm) and application scenario. The data collection method is one beacon to one scanner that measures the RSSI data at the set distance, 1000 samples for each measurement point (refer to Figure 7).

**Figure 13:**
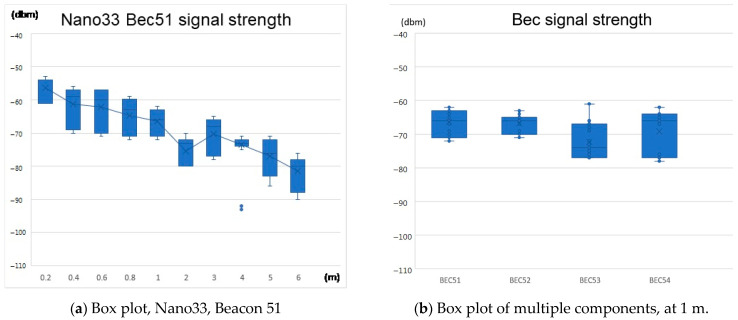
Signal strength box plots.

If we look at one of the RSSI measurement results (Nano 33, BEC51), it is shown as a box plot in Figure 13a. The strength of the RSSI signal gradually attenuates from 0.2 m to 6 m. At 4 m, the box plot quartile value (upper and lower combined) is narrower; this effect could be attributed to the unexpected positive signal performance at this distance or to remove several outliers. Thus, from a relatively simplified perspective, Figure 13 has constituted an RSSI descending trend. Figure 13b shows the RSSI signal strength values of #51, 52, 53, and 54 measured at 1 m; this sequence of the box plot displays the differences in individual components.

- Validation in distance

This section explains the actual measurement conversion error when using the data presented in Table 7. In the previous explanation, the regression coefficients, box plot, and filtered outliers were utilized to moderate the RSSI–distance conversion to optimize the value.

The measured RSSI of the beacon (BEC51) was converted into the distance using the signal propagation model, and the results are shown in Table 8. The accuracy remained below 10 cm within one meter, but the error gradually increased to more than 1 m as the distance between the beacons and scanner increased as well. The results showed the optimum RSSI–distance converting range can be 0.6–1 m.

It is vital to notice that each device has its own particular coefficient calculation for this research due to the difference in signal propagation and RSSI conversion, as shown in Figure 13b. Further details are explained in the Discussion section.

### 3.2. Multi-Beacon (Result)

Location positioning with Trilateration requires three reference points (beacons) minimum. According to the previous RSSI–distance analysis, RSSI data filtering is mandatory to improve the positioning accuracy. The following subsection explains the point selection methodology (multi-point or top three points). Additionally, the positioning accuracy in the Common setup and Intensive setup in the classroom will be discussed as well.

#### 3.2.1. Point Selection

The general idea for positioning calculations is to remove the weaker points first, using the threshold of −94 dBm, as defined previously. There are two point selection modes: multi-point and top-three points.

**Figure 14:**
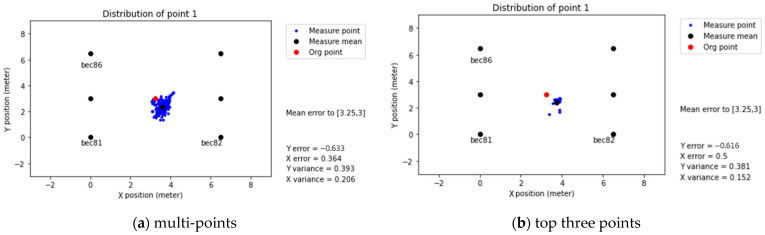
Comparison of the points section in the positioning accuracy.

In Figure 14a, the six beacons are used to create the prediction location, point 1, without considering signal strength limitation (multi-point). On the other hand, Figure 14b presents the prediction from the top three points. The results show that the multi-point alternative could have better position accuracy in comparison to the top-three points.

It is possible to notice that the top-three point positioning method generates data loss, reduces the number of positioning points, and increases its data offset, resulting in a higher overall error, even after applying the Least Squares Method (LSM).

In contrast, the data amount is relatively large for multi-point positioning. However, after using LSM, the standard deviation of the data is significant. In this case, the error of each receiver was added to the calculations, affecting the standard deviation error. Therefore, to improve the performance of the system, a threshold must be selected.

#### 3.2.2. Common Setup

**Figure 15:**
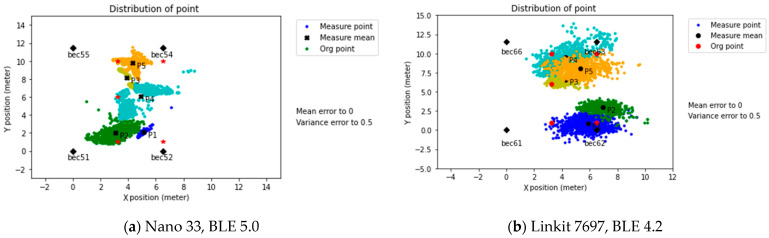
Common setup and predicted positioning.

The scanner moved along a path of set points (P1–P5) to collect data in this setup. The orientation among the beacons and scanners affected the signal strength of our expectations. In Figure 15, the red star is the actual location of the scanner, and the black cross represents the predicted point in the classroom.

- Arduino Nano 33 in the classroom

According to Table 9, the actual data distribution standard deviation was about 1 m; the X-Y position error was about ±2 m. The error increased when the scanner was near the wall, as shown in Figure 15.

#### 3.2.3. Intensive Setup

**Figure 16:**
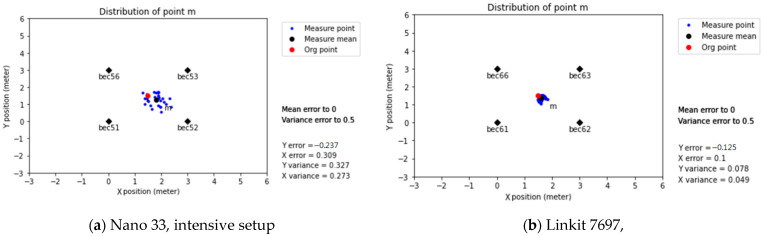
Intensive setup and predicted positioning.

Referring to the previous experimental results in Section 3.2.2, the errors in the positioning increased when the scanner was close to the wall. That caused the measured positioning point to shift away. The receiver was located in a new location in the experimental field to observe the wall influence, and four new beacon signals were measured at the central position. The results showed that the offset error and dispersion degree of the positioning point was significantly lower, improving the prediction to a combined value of x = 8.9 cm, y = 4.5 cm, as shown in Figure 16b.

Establishing a measurement distance equal to or less than three meters helped provide a stable RSSI signal and significantly improved the distance prediction. Additionally, this indirectly proves that terrain factors such as walls do interfere with the signal.

### 3.3. Diverse Direction in Geometric Spatial (3D Results)

The measurement conditions are different from the previous sections, increasing the beacons, distance, and environment conditions (workshop). The orientation between emitter and receiver has a diverse direction in geometric space, close to the measurement position established in Figure 6. This measurement environment could generate radio wave reflection due to the presence of CNC machinery nearby. The distance between the scanner and the different beacons was approximately two meters, as shown in Figure 10. This setup was used for both models, BLE 5.0 and BLE 4.2.

Trilateration and Modification coefficients were applied for the position calculations. Beacon BLE 5.0 (Nano 33) and Beacon BLE 4.2 (Linkit7697) were compared with and without a modification coefficient in the workshop manufacturing environment.

#### 3.3.1. Workshop, BLE 5.0 (Nano 33)

The predicted positioning of BLE 5.0 (Nano 33) was divided into two parts, with and without modification coefficients. The positioning results without a modification coefficient had significant mean and variance errors. In addition, the distribution was irregular.

- Predicted positioning (without Modification coefficients)

**Figure 17:**
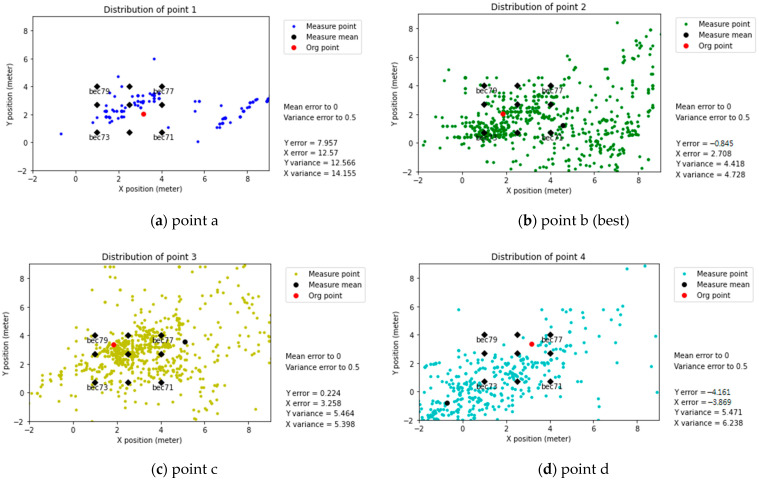
Predicted positioning (without modification coefficients), BLE 5.0 (Nano 33).

**Figure 18:**
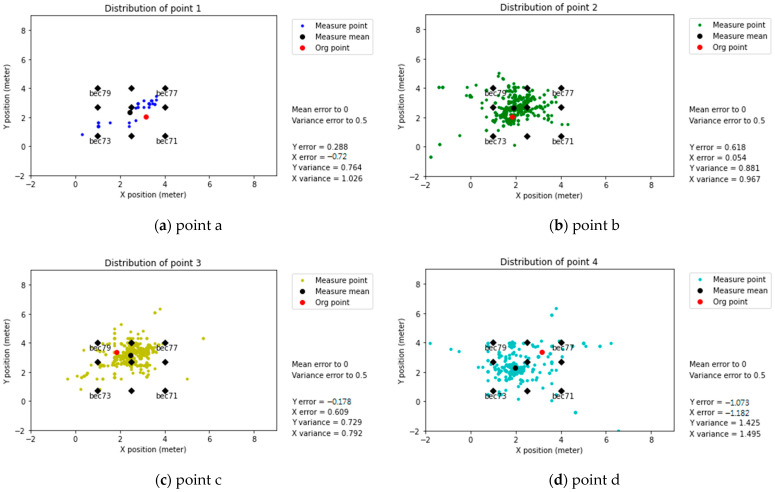
Predicted positioning (with modification coefficients), BLE 5.0 (Nano 33).

Figure 17 shows points a, b, c, and d (present as 1, 2, 3, and 4 in the plots) and their predicted values projected on the x-y coordinates. Figure 18a predicted positioning fluctuates between 5 and 6 m. In comparison, the position data presented in Figure 17b–d can be up to 8 m in range. However, based on the mean value and its original points, the best accuracy measure point is at point b (point 2 in the plot). For example, regarding Figure 17b (Point b), the X- and Y-axis errors are 2.468 and −0.551, and the variances are 3.569 and 3.585, respectively.

- Predicted positioning (with Modification coefficients)

It is possible to appreciate the effect of applying modification coefficients. The mean error and variance error decrease and the predicted positions become more concentrated, as shown in Figure 18. In this case, for points a, b, c, and d, the error is approximately between 0.5 and 1.5 m.

It is vital to notice outliers outside the perimeter of the beacons, as shown in Figure 17c (predicted positioning without modification coefficients). Figure 18c presents the same point, applying modification coefficients. In this particular case, the significant amount of outlier data affected the performance of the modification coefficients, with a minor effect on the error and a significant reduction of variance.

#### 3.3.2. Workshop, BLE 4.2 (Linkit 7697)

In this subsection, the predicted position error before applying modification coefficients is less than BLE 5.0. However, this phenomenon could be attributed to the difference in transmitting power between BLE 5.0 and BLE 4.2 mentioned in Table 2.

- Predicted positioning (without Modification coefficients)

**Figure 19:**
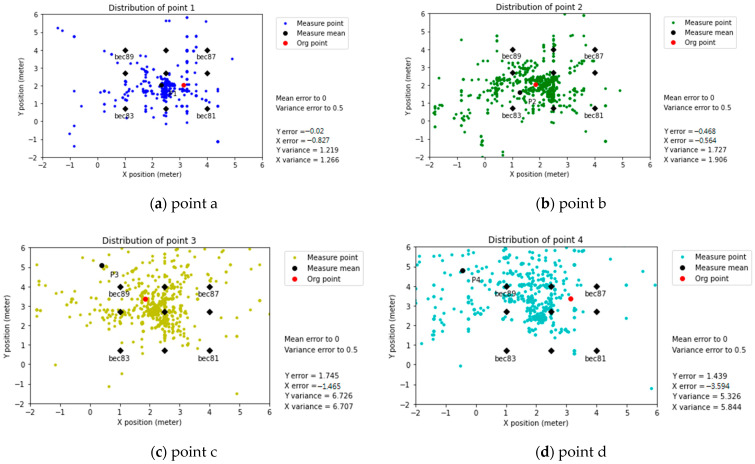
Predicted positioning (without modification coefficients), BLE 4.2 (Linkit7697).

Regarding the influence of work environment situations, physical refraction was present in the results of this experiment. It was possible to observe that the predicted positioning points of a and b were more concentrated than c and d. In the setup, c and d were closer to the wall, which increased the X error and Y error, as shown in Figure 19.

- Predicted positioning (with coefficients modification)

**Figure 20:**
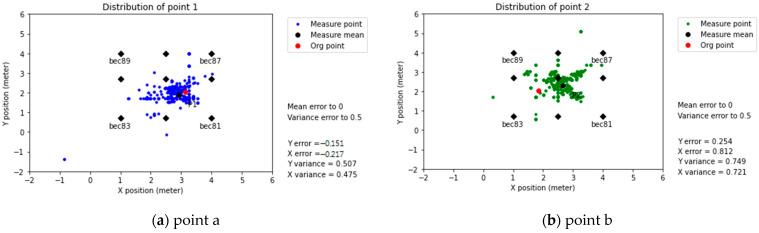

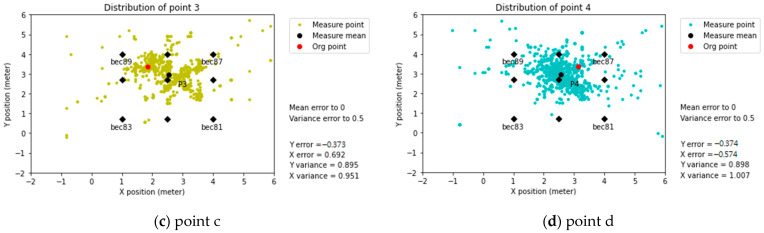
Predicted positioning (with modification coefficients), BLE 4.2 (Linkit7697).

After applying modification coefficients, the BLE 4.2 positioning results had an average error slightly smaller than BLE 5.0, fluctuating in the interval of 0.5–1 m, as shown in Figure 20. The standard deviation error was about the same for both devices (BLE 4.2 and BLE 5.0). However, the influence of the radio wave refraction seemed to affect BLE 4.2 more due to the predicted positioning dispersion, as shown in Figure 20d.

### 3.4. Centroid Deviation (Interpretation)

From the previous predicted positioning figures, it was possible to observe a deviation from the measured mean to the centroid (measurement point). The perceived deviation from the centroid can be caused for the three main factors: physical space, type of beacon (4.2 or 5.0), and noise.

Firstly, the physical space can significantly affect the distance prediction accuracy; from the results of the Multi-Beacon Measurement in the classroom, it was possible to observe the effect of walls in predicting the distance, with a X-Y position error of ±2 m, as shown in Figure 15. When the system setting changed from the Common to Intensive setup, the error reduced to 8–10 cm, as shown in Figure 16. This effect was also present in the workshop environment, where the points close to the wall exhibited higher errors, as shown in Figure 19c,d.

Secondly, regardless of the environmental conditions, the BLE 5.0 signal tended to perform better than its predecessor. The predicted positioning tended to be more concentrated when the modification coefficient theorem was applied, as shown in Figure 18.

Finally, the influence of noise could affect the positioning results. Even if the modification coefficient method could correct the results while decreasing its average error, the effect of noise persisted. BLE 4.2 errors were lower in high-refraction environments (workshop), as shown in Figure 19. In contrast, BLE 5.0 provided a more accurate predicting position among the low-refraction environments (classroom), as shown in Figure 15a. However, after applying the modification coefficient, the effect of noise persisted, affecting the position predictions of BLE 5.0, as shown in Figure 18d.

## 4. Discussion

In the Results section, many plots illustrated the actual measurement situation in the field, either of a less-noisy environment (classroom) or manufacturing site (workshop). These practical RSSI signals helped us to understand the reception status of the beacons and scanner. More than 1.6 million samples were collected for this experiment, generating over 135 MB of data in JSON format. When collecting the beacon data, all the signals were indeed received, but some messages were missed due to the receiver sensitivity (Table 2); in this case, the values with RSSI lower than −94 dBm were lost. Besides the quantity of the data, it is essential to consider some relevant topics such as the sampling rate or antenna polarity.

### 4.1. Sampling Rate

Sample collection is the primary purpose of the scanner, harvesting the signal of each beacon and packing, transporting, and delivering it to the database to analyze the behavior of the whole Indoor Positioning System. When collecting samples, it is crucial to understand if the whole system can satisfy the requirements from the Nyquist rate, which states that the frequency of sampling must be double the base frequency to obtain a discrete time sequence free of distortion (aliasing).

$$\;f_{s}>2B$$

The experiment conducted used different types of hardware as the beacons (Nano33 and MT7697), and the emitter interval of both models was established as 100 ms (10 Hz). Based on the Nyquist rate, the scanner must achieve a sampling rate frequency of 20 Hz or higher $\left(f_{s}\right)$.

### 4.2. System Latency

Internally, the microcontroller (MT7697) performed the whole datalink layer process in 20 ms. Therefore, the Quality of Service (QoS) of the system was zero (QoS 0). Under these circumstances, MQTT generated a delay equal to or quicker than 5 ms, according to the Benchmark for MQTT brokers provided by Scalagent. Based on practical experience, the total latency of the system was 32 ms. Therefore, the delay caused by the Network (IPv6) and Transportation (TCP) can be induced as 7 ms.

With a sampling rate of 31 Hz $\left(f_{s}\right)$, it is possible to guarantee a signal without sampling distortion (aliasing), satisfying the Nyquist rate minimum frequency (20 Hz).

### 4.3. Antenna Polarity

**Figure 21:**
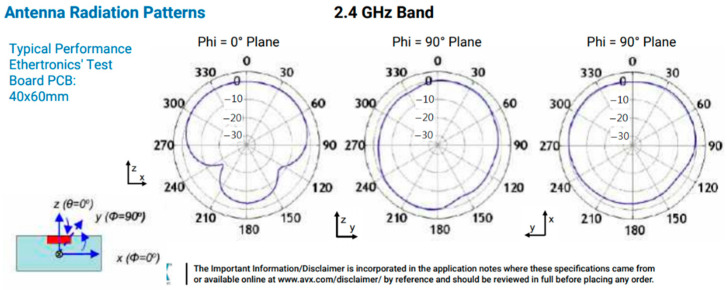
Antenna radiation pattern (AVX).

The sensitivity of the antenna fluctuates widely due to its design; according to the antenna’s orientation, the signal will suffer attenuation caused by the physical effects of the antenna related to its topology. In this study, the ceramic antenna was 3 × 1.5 mm; the following image represents the antenna radiation pattern, according to AVX reference [41], as shown in Figure 21.

It is possible to observe the attenuation that the ceramic antenna suffers 180 degrees (backward). This fact corresponds to the practical observations noted in Table 4 (Orientation).

## 5. Conclusions

Based on the results from the previous experiments, our team concluded that the key factor in developing a reliable indoor positioning system is the signal measurement quality. Collecting stable RSSI signals and improving the architecture should be the priority due to the considerable influence of signal refraction (noise).

Firstly, hardware selection greatly influenced the final results. For BLE 4.2, the influence of the radio wave refraction affected the predicted positioning, as shown in Figure 20d. BLE 5.0 presented a higher signal stability and better performance in lower-interference environments, as shown in Figure 15a. Multiple factors affected the RSSI, such as the device performance (Table 2), antenna direction (Figure 3), and radio wave refraction (Figure 17).

Secondly, using the RSSI regression coefficient cannot guarantee the system’s accuracy due to outliers and the lack of data interpretation (Table 6). For this topic, the use of modification coefficients helped reduce the data variance, as shown in Figure 20. In addition, the use of an intensive beacon set up in a low interference environment improved the accuracy effectively, as shown in Figure 16.

Finally, we aim to combine the methodology developed in this research with new indoor location technologies, such as extended Kalman filters or the Angle of Arrival (AOA) (introduced in BLE 5.2), for our future research development.

## Figures and Tables

**Table 1:** The portions in the practice of the BLE measurements.

Components	Measurement	Analysis Methods
Specification	RSSI–distance conversion	Signal propagation
Interconnection	Multi-beacon in-plane	Trilateration
	Diverse directions in spatial	Modification coefficients
		Kalman Filtering *

* Kalman Filtering listed as a further topic, but the relevant analysis results are not included in this report.

**Table 2:** Specifications of the components (BLE 5.0 and BLE 4.2).

Model	BLE Version	Transmitting	Receiving	Remark
Arduino Nano 33	BLE 5.0 ^1^	−20 to +8 dBm	−103 dBm (long range) −95 dBm (low energy)	Beacon
Linkit 7697	BLE 4.2 ^2^	−21 to +9 dBm	−94 dBm	Beacon Scanner

^1^ Nordic nRF52840 chipset: https://content.arduino.cc/assets/Nano_BLE_MCU-nRF52840_PS_v1.1.pdf (accessed on 28 July 2021). ^2^ ACSIP wrtnode7 chipset: http://www.acsip.com.tw/index.php?action=technical&p=2 (accessed on 28 July 2021).

**Table 3:** Antenna orientation influence on the RSSI.

Distance	Face-to-Face		Back-to-Back	
m	RSSI ^1^	Var	RSSI	Var
0.2	−56	15.64	−79	14.51
0.4	−63	6.68	−85	18.44
0.6	−66	0.77	−87	34.54
0.8	−66	3.57	−82	19.90
1	−66	17.26	−85	28.51
2	−72	19.34	−86	19.44
3	−73	3.09	−93	1.82

^1^ RSSI: Received Signal Strength Index, unit: dBm; mean value (1000 samples).

**Table 4:** Components numbering (BLE 5.0 and BLE 4.2).

Model	BLE 5.0	BLE 4.2	Location
Arduino Nano 33	#51–56	-	Classroom ^1^
Linkit 7697	-	#61–66	Classroom
Arduino Nano 33	#71–79	-	Workshop ^2^
Linkit 7697	-	#81–89	Workshop

^1^ Classroom: less noisy environment (refer to Figure 5c). ^2^ Workshop: general factory noise (refer to Figure 6c).

**Table 5:** Interconnection model and protocols.

OSI Layer ^1^	IoT Protocol	Component
Application	MQTT broker ^2^	Server
Transport	TCP	Router
Network	IPv4, IPv6	Access Point
Datalink	BLE, MAC, 802.11 b/g/n (Wi-Fi)	Microcontroller

^1^ Open Systems Interconnection model (OSI model), https://en.wikipedia.org/wiki/OSI_model (accessed on 28 July 2021). ^2^ MQTT broker, https://en.wikipedia.org/wiki/MQTT (accessed on 28 July 2021).

**Table 6:** The distance between the beacons and measurement point (scanner).

Beacon No.^1^	Point a	Point b	Point c	Point d
#71 (#81)	2.26 m	-	-	-
#72 (#82)	2.20 m	2.20 m	-	-
#73 (#81)	-	2.26 m	-	-
#74 (#81)	1.92 m	-	-	1.92 m
#75 (#81)	1.84 m	1.84 m	1.85 m	1.85 m
#76 (#86)	-	1.92 m	1.92 m	-
#77 (#87)	-	-	-	1.92 m
#78 (#88)	-	-	1.85 m	1.85 m
#79 (#89)	-	-	1.92 m	-

^1^ Arduino nano33 (#71–#79) and Linkit 7697 (#81–#89).

**Table 7:** RSSI/distance conversion table.

Beacon/Distance ^1^	0.2 m	0.4 m	0.6 m	0.8 m	1.0 m	2.0 m	3.0 m	4.0 m	5.0 m	6.0 m	7.0 m	8.0 m	9.0 m	10 m
Arduino Nano 33 dBm ^2^	−56	−63	−66	−66	−66	−72	−73	−73	−77	−85	−84	−85	−83	−86
Linkit 7697 dBm	−55	−62	−61	−65	−68	−74	−69	−76	−74	−79	−79	−79	−79	−83

^1^ Distance unit: meter. ^2^ RSSI: Received Signal Strength Index, unit: dBm. The RSSI value was acquired based on its median with 1000 samples.

**Table 8:** Distance prediction and error.

Device	Actual Distance (m)	Predicted Distance ^1^	Error (m)	Var
Bec #51	0.2	0.263	0.063	0.024
	0.4	0.518	0.118	0.260
	0.6	0.603	0.003	0.312
	0.8	0.880	0.080	0.427
	1.0	1.098	0.098	0.203
	2.0	1.373	−0.627	1.470
	3.0	1.863	−1.137	1.158
	4.0	2.734	−1.266	2.726
	5.0	4.344	−0.656	3.263
	6.0	7.283	1.283	7.113

^1^ Predicted distance: median value calculated based on its regression coefficients (1000 samples).

**Table 9:** Mean positioning error and variance, Nano 33, BLE 5.0.

	Location	Point 1	Point 2	Point 3	Point 4	Point 5
Notification						
Y error		1.144	1.018	2.18	−3.876	−0.163
X error		−1.365	−0.152	0.643	1.678	−2.187
Y variance		0.51	0.667	0.762	1.159	0.524
X variance		0.392	0.813	0.363	0.937	0.501

## Data Availability

The data presented in this study are available on request from the corresponding author. The data are not publicly available due to its use on future research activities.
